# Brain Endothelial Cells Regulate Glucagon-Like Peptide 1 Entry Into the Brain via a Receptor-Mediated Process

**DOI:** 10.3389/fphys.2020.00555

**Published:** 2020-05-29

**Authors:** Zhuo Fu, Liying Gong, Jia Liu, Jing Wu, Eugene J. Barrett, Kevin W. Aylor, Zhenqi Liu

**Affiliations:** ^1^Division of Endocrinology and Metabolism, Department of Medicine, University of Virginia Health System, Charlottesville, VA, United States; ^2^Department of Pharmacology, The Third Xiangya Hospital of Central South University, Changsha, China; ^3^Department of Endocrinology, Xiangya Hospital, Central South University, Changsha, China

**Keywords:** Glucagon-like peptide 1, blood brain barrier, endothelial cells, microvasculature, protein kinase A

## Abstract

Glucagon-like peptide 1 (GLP-1) in addition to regulating glucose-dependent insulin and glucagon secretion exerts anorexic and neuroprotective effects. While brain-derived GLP-1 may participate in these central actions, evidence suggests that peripherally derived GLP-1 plays an important role and GLP-1 analogs are known to cross the blood brain barrier. To define the role of brain microvascular endothelial cells in GLP-1 entry into the brain, we infused labeled GLP-1 or exendin-4 into rats intravenously and examined their appearance and protein kinase A activities in various brain regions. We also studied the role of endothelial cell GLP-1 receptor and its signaling in endothelial cell uptake and transport of GLP-1. Systemically infused labeled GLP-1 or exendin-4 appeared rapidly in various brain regions and this was associated with increased protein kinase A activity in these brain regions. Pretreatment with GLP-1 receptor antagonist reduced labeled GLP-1 or exendin-4 enrichment in the brain. Sub-diaphragmatic vagus nerve resection did not alter GLP-1-mediated increases in protein kinase A activity in the brain. Rat brain microvascular endothelial cells rapidly took up labeled GLP-1 and this was blunted by either GLP-1 receptor antagonism or protein kinase A inhibition but enhanced through adenylyl cyclase activation. Using an artificially assembled blood brain barrier consisting of endothelial and astrocyte layers, we found that labeled GLP-1 time-dependently crossed the barrier and the presence of GLP-1 receptor antagonist blunted this transit. We conclude that GLP-1 crosses the blood brain barrier through active trans-endothelial transport which requires GLP-1 receptor binding and activation.

## Introduction

Glucagon-like peptide (GLP-1) regulates glucose-dependent insulin and glucagon secretion. It acts by binding to and activating GLP-1 receptors which are expressed in many tissues including islets, heart, vasculature, liver and central nervous system (Richards et al., 2014). GLP-1 also has extra-pancreatic effects on satiety, food intake, energy metabolism and cardiovascular system and GLP-1 receptor agonism has become a mainstay in the management of type 2 diabetes mellitus, particularly in patients with atherosclerotic disease (American Diabetes Association, 2018).

Many of GLP-1’s effects are mediated via actions on various parts of the brain. Manipulation of brain GLP-1 action clearly alters glucose metabolism and insulin sensitivity but brain GLP-1 receptors are not needed for normal glucose control (Cabou et al., 2011; Sandoval and Sisley, 2015). GLP-1 receptors are expressed in various regions of human, primate, and rodent brains including cerebellum, cortex, hippocampus, and hypothalamus (Alvarez et al., 2005; Hamilton and Hölscher, 2009; Cork et al., 2015; Heppner et al., 2015). Accumulating evidence suggests that brain GLP-1 receptors mediate GLP-1’s neuroprotection and many autonomic and neuroendocrine functions (Imeryüz et al., 1997; Larsen et al., 1997; During et al., 2003; Kinzig et al., 2003). Brain derived GLP-1, produced in pre-proglucagon neurons located in the nucleus of the solitary tract and projecting to numerous brain regions, has been shown to control a range of feeding responses and energy balance (Trapp and Richards, 2013). Moreover, peripherally administered GLP-1 analogs, such as liraglutide and Val(8)GLP-1, can reduce amyloid plaque formation, prevent memory impairment and synapse loss in hippocampus, and rescue synaptic plasticity in a mouse model of Alzheimer disease (McClean et al., 2011; Gengler et al., 2012) and protect against Aβ1-40-induced impairment of hippocampal late-phase long-term potentiation and spatial learning in rats (Wang et al., 2010).

For circulating GLP-1 (or GLP-1 analogs) to act on neurons, it must first cross the blood brain barrier (BBB) to reach the brain interstitial fluid compartment. The BBB not only serves as a barrier but also provides endothelial exchange surface area for substrates, such as oxygen, nutrients, and hormones to access neurons and astrocytes. GLP-1 analogs injected peripherally appear to rapidly (within 5 min) enter the brain in a time-dependent fashion (Kastin and Akerstrom, 2003). Whether and how this process is regulated remain unknown.

Peptides cross the BBB via transcellular vesicular pathways that may be either constitutive or regulated. The entry of ^125^I-exendin-4 into the brain is inhibited by high concentrations of unlabeled exendin-4, suggesting a saturable process (Kastin and Akerstrom, 2003). This is similar to leptin (Banks et al., 1996) and insulin (Gray et al., 2014; Meijer et al., 2016), two appetite regulating hormones secreted peripherally that enter into the brain.

Vascular endothelium expresses abundant GLP-1 receptors (Nystrom et al., 2004) which mediate arteriolar dilation and regulate tissue perfusion (Drucker Daniel, 2016). In the current study, we hypothesized that brain endothelium regulates GLP-1 (and GLP-1 analog) entry into the brain. Our results indicate that vascular endothelial cells actively take up and transport GLP-1 across the BBB and these actions are dependent on its binding to GLP-1 receptors and the activation of adenylyl cyclase.

## Materials and Methods

### Animals

Healthy adult male Sprague-Dawley rats (350–400 g), subdiaphragmatic vagotomized and sham-operated adult male Sprague-Dawley rats (∼200 g) were all purchased from Charles River Laboratories (Wilmington, MA, United States). They were fed with a standard chow diet (protein 28 kcal%, carbohydrate 60 kcal%, and fat 12 kcal%), housed at 22 ± 2°C on a 12 h light-dark cycle and had access to water *ad libitum*. The completeness of vagotomy was confirmed using fluorogold staining as described in the literature (Jordi et al., 2013). Briefly, 4 days after an intraperitoneal (i.p.) injection of fluorogold (1 mg, Sigma-Aldrich Corp., St. Louis, MO, United States), vagotomized or sham-operated rat brains were collected and fixed in formalin after rats were sacrificed and brain sections were visualized using a fluorescence microscope (Olympus, Japan). Body weight and daily food intake were recorded during this time period.

Prior to each infusion study, rats were fasted overnight, and anesthetized with thiobutabarbital (Inactin, i.p. 120 mg/kg, Sigma-Aldrich, St. Louis, MO, United States). They were placed in a supine position on a heating pad to ensure euthermia and intubated to maintain a patent airway. The right jugular vein and left carotid artery were cannulated with polyethylene tubing (PE-50, Fisher Scientific, Newark, DE, United States) and each rat was allowed a 30–45 min to ensure hemodynamic stability and a stable level of anesthesia before they were subject to various infusions, as detailed below.

### Circulating GLP-1 Uptake by the Brain

**FIGURE 1:**
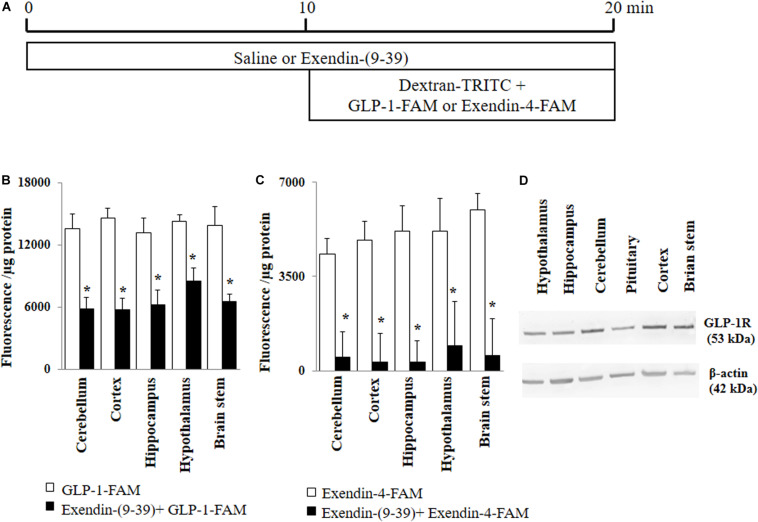
GLP-1 and GLP-1 analog exendin-4 enter various brain regions via GLP-1 receptor-mediated process. **(A)** Protocol. Each rat received saline or exendin-(9-39) (30 nmol/kg/min) for 10 min and then dextran-TRITC (30 pmol/kg/min) + GLP-1-FAM (30 pmol/kg/min) or exendin-4-FAM (30 pmol/kg/min) for 10 min via a carotid artery catheter. Brain tissues were harvested after systemic flush with saline. **(B)** GLP-1-FAM uptake by the brain (*n* = 7, *p < 0.05 vs. GLP-1-FAM alone, Student’s *t* test). **(C)** Exendin-4-FAM uptake by the brain (*n* = 6, *p < 0.05 vs. Exendin-4-FAM alone, Student’s *t* test). **(D)** GLP-1 receptor protein expression in different regions of the brain (Western blot). GLP-1R, GLP-1 receptor.

Rats received a systemic infusion of either saline or exendin-(9-39) (GLP-1 receptor antagonist, 30 nmol/kg/min, Abcam, Cambridge, United Kingdom) via the jugular vein catheter for 20 min (Figure 1A). In the last 10 min, rats also received 30 pmol/kg/min of either GLP-1-FAM (AnaSpec, Fremont, CA, United States) or exendin-4-FAM (AnaSpec, Fremont, CA, United States) together with Dextran-TRITC (Sigma-Aldrich, St. Louis, MO, United States) (70 kD, vascular space marker, 30 pmol/kg/min). The infusion doses were based on our prior studies in rats that GLP-1 at 30 pmol/kg/min potently increased muscle microvascular perfusion and glucose use (Chai et al., 2012, 2014; Dong et al., 2013). After collecting plasma samples, rats were sacrificed via anesthetic overdose and various brain regions (cerebellum, cortex, hippocampus, hypothalamus, and brain stem) collected, homogenized and lysed in lysis buffer. Fluorescence from TRITC and FAM were measured in both plasma and brain lysates. Protein concentrations were determined in the brain lysates. Brain uptake of GLP-1-FAM and exendin-4-FAM were calculated using the following formula:

$$\begin{array}{rcl} & & \frac{\mathrm{Tissue}\;\mathrm{fluorescence}\;[\mathrm{FAM}]}{\mathrm{Tissue}\;\mathrm{weight}\;(\mu g)}-\frac{\mathrm{Tissue}\frac{\mathrm{Fluorescence}\;[\mathrm{TRITC}]}{\mathrm{Weight}}(\mu g)}{\mathrm{Plasma}\frac{\mathrm{Fluorescence}\;[\mathrm{TRITC}]}{200}(\mu L)}\\ & & \;\times \;\mathrm{Plasma}\;\mathrm{Fluorescence}\;[\mathrm{FAM}]/200\mu L \end{array}$$

### Brain Microvessel Isolation and Brain Tissue Preparation

Each rat received a systemic infusion of either saline or GLP-1 (Abcam, Cambridge, United Kingdom) (30 pmol/kg/min) via the jugular vein catheter for 10 min. Plasma samples were then collected and rats sacrificed via anesthetic overdose. Cerebral spinal fluid (CSF) and various brain regions (cerebellum, cortex, hippocampus, hypothalamus, and brain stem) were collected for further assays. Cerebral microvessels were isolated as described (Austin et al., 2010). Briefly, brain tissue was disrupted with a razor blade followed by passing through an 18G needle. Vessel fragments were harvested using dextran-gradient centrifugation, then collected on a 20 μm pore filter, washed to remove cell debris, and reconstituted in buffer. This was followed by passing through a 100 μm pore size filter. Brain vessels that can pass through the 100 μm filter were considered as cerebral microvessel and used for protein kinase A (PKA) activity assay. Cerebral microvessels were verified by Lectin I (Vector Lab, Burlingame, CA, United States) and hematoxylin (Thermo Fisher Scientific Waltham, MA, United States) staining.

### Determination of GLP-1 Concentrations and PKA Activity

All plasma and CSF samples collected during the study were frozen immediately and stored at −80°C until further analysis of GLP-1 concentration using a total GLP-1 ELISA kit (Merck Millipore, Billerica, MA, United States) (Knezovic et al., 2018). To measure PKA activity in brain tissue, cerebral microvessels, or cultured cells, samples were homogenized and lysed in PKA lysis buffer and activity determined using a Pep Tag^®^ non-radioactive protein kinase assay kit (Promega, Madison, WI, United States) (Christian et al., 2011). Phosphorylated peptide migrates toward the cathode while nonphosphorylated peptide migrates toward the anode. Commercial positive and negative controls were used to confirm the band specificity.

### Endothelial Cell Uptake of GLP-1 and Exendin-4

Rat brain microvascular endothelial cells (RBMECs; Cell Applications, San Diego, CA, United States) were cultured on a cover slip in rat brain endothelial cell growth medium. After reaching confluency, cells were pre-treated with vehicle (PSS buffer), exendin 9-39 (10 nM) or H89 (Sigma-Aldrich, St. Louis, MO, United States) (10 μM) for 30 min or forskolin (Sigma-Aldrich, St. Louis, MO, United States) (10 μM) for 5 min. Cells were then incubated with GLP-1-FAM at either 10, 100 or 1,000 pM for 3 min at 37°C. DAPI was used to stain the nucleus. After fixation and washing three times with cold TBST (5 min each time), cell images were captured using confocal microscope and fluorescence intensity measured using ImageJ software^1^. FAM signal from surface binding of FAM was examined by incubating cells with GLP-1-FAM at 4 or 37°C for 3 min, or free FAM (10, 100, and 1,000 pM) at 37°C for 3 min.

### Trans-Endothelial GLP-1 Transport

The trans-endothelial GLP-1 transport was examined using a Transwell system that mimics the BBB in that RBMECs were co-cultured with astrocytes as illustrated in Figure 7A and previously described (Gaillard et al., 2001). Briefly, astrocytes (Thermo Fisher Scientific, Waltham, MA, United States) were seeded on the bottom and RBMECs were then seeded onto the top of the Transwell insert (6.5 mm diameter, 1 μm pore size, polyester membrane; Corning, Corning, NY, United States). Trans-endothelial electrical resistance was monitored using a volt-ohmmeter (EVOM; WPI, Sarasota, FL, United States) and EndOhm chamber (EndOhm; WPI). When the trans-endothelial electrical resistance reached its peak, the insert with double cell layers was washed twice at 37°C with PBS buffer. After pre-treatment of the cells with either vehicle (PBS buffer) or exendin-(9-39) (100 nM) for 10 min, 100 pM GLP-1-FAM or inulin-FITC (Sigma-Aldrich, St. Louis, MO, United States) was loaded into the upper chamber in the presence of DPP4 inhibitor (MilliporeSigma, Burlington, MA, United States) with or without exendin-(9-39). The amount of GLP-1-FAM or inulin-FITC transported to the lower chamber was determined by measuring FAM and FITC fluorescence in the lower chamber at 5, 10, and 30 min. Inulin-FITC transport was used to correct for paracellular transport and the amount of trans-endothelial transport of GLP-1-FAM was calculated using the formula:

$$\begin{array}{c} \mathrm{Transcellular}\;\mathrm{transport}\;(\%)\\ =\frac{\mathrm{Fluorescence}\;[\mathrm{FAM}]}{\mathrm{Reference}\;[\mathrm{FAM}]}\times 100-\frac{\mathrm{Fluorescence}\;[\mathrm{FITC}]}{\mathrm{Reference}\;[\mathrm{FITC}]}\times 100 \end{array}$$

Reference refers to fluorescence in the lower chamber at the specified time point when the Transwell inserts had no cells seeded. To determine if this trans-endothelial transport is dose-dependent, 10, 100, and 1,000 pM GLP-1-FAM or inulin-FITC (with DDP-4 inhibitor) was loaded into the upper chamber, the amount of GLP-1-FAM or inulin-FITC in the lower chamber was determined at 10 min, and trans-endothelial transport of GLP-1 was calculated.

### Western Blot and Immunohistochemistry Determination of GLP-1 Receptor Expression

GLP-1 receptor expression in various brain regions, vasculature, and cultured astrocytes and RBMECs was determined using Western blot. Primary antibodies against GLP-1 receptor and β-actin were obtained from Abcam (Cambridge, MA, United States). All blots were developed using enhanced chemiluminescence (GE Healthcare Bio-Sciences Corp, Piscataway, NJ, United States). Autoradiographic films were scanned densitometrically and quantified using ImageJ (NIH^1^). Both GLP-1 receptor and β-actin density was quantified and the ratios of GLP-1 receptor to β-actin protein density calculated.

RBMECs cultured on a cover slip were treated with attachment factor solution (Cell Applications, San Diego, CA, United States), fixed in 4% paraformaldehyde for 10 min at room temperature and incubated with or without primary antibody against GLP-1 receptor (Abcam, Cambridge, MA, United States) overnight at 4°C. The slides were then incubated with HRP conjugated secondary antibody (Abcam, Cambridge, MA, United States), followed by a counter staining with hematoxylin (Thermo Fisher Scientific Waltham, MA, United States), and mounted with mounting medium before subject to examinations using a light microscope (Olympus, Japan).

### Quantification of Microvascular Perfusion in Rat Hippocampus and Skeletal Muscle

After a 30–45 min baseline period to ensure hemodynamic stability and a stable level of anesthesia, baseline microvascular blood volume (MBV) and microvascular flow velocity (MFV), in the hippocampus and skeletal muscle were determined using contrast-enhanced ultrasound (CEU), as described previously (Chai et al., 2010; Fu et al., 2013, 2017). In brief, microbubbles (Definity; Lantheus Medical Imaging) were diluted with normal saline (1:10 vol:vol) and infused intravenously at a rate of 0.8 mL/h. Once the systemic microbubble concentrations reached steady state (∼6 min), high-power pulse-inversion ultrasound (HDI-5000; Philips Ultrasound) was used to image the microbubble signals in the hippocampus and the proximal adductor muscle group (adductor magnus and semimembranosus). Intermittent imaging was performed at pulsing intervals ranging from 0.5 to 10 s. At least three images were acquired at each pulsing interval, and the images obtained at 0.5 s were used as background. Pulsing interval vs. background-subtracted video intensity was fitted to the function *y* = *A* (1 − e^−β*t*^, with *y* being video intensity at pulsing interval *t. A* as the plateau video intensity measures MBV and β represents the rate of microvascular filling (i.e., MFV). Microvascular blood flow (MBF) was calculated by multiplying MBV and MFV. All CEU images were analyzed using the QLAB software (Philips Medical Systems, Andover, MA, United States).

After the baseline hippocampal and muscle microvascular parameters were obtained, rats then received a continuous intravenous infusion of either saline or GLP-1 (30 pmol/kg/min) for 120 min. Hippocampal microvascular parameters (MBV, MFV, and MBF) were again determined every 30 min and muscle microvascular parameters at 120 min using CEU. Throughout the study, mean arterial blood pressure (MAP) was monitored via a sensor connected to the carotid catheter (Harvard Apparatus, Holliston, MA, United States, and AD Instruments, Inc., Colorado Springs, CO, United States). Inactin was infused at a variable rate to maintain steady levels of anesthesia and blood pressure throughout the study. Carotid arterial blood glucose concentrations were determined every 30 min using a Contour Next glucometer (BAYER). At the end of each study the rat was euthanized by an overdose of inactin.

### Glucose Tolerance Test

Glucose tolerance test was used to confirm that GLP-1-FAM has a biological efficacy similar to GLP-1 (Supplementary Figure 1). Briefly, overnight fasted rats were anesthetized with thiobutabarbital (Inactin, i.p. 120 mg/kg). After 30 min of stabilization, the rat received a continuous i.v. infusion of GLP-1-FAM (30 pmol/kg/min), GLP-1(30 pmol/kg/min) or saline or an i.v. injection of liraglutide (30 pmol/kg) along with a bolus i.v. injection of glucose (0.5 g/kg). Carotid arterial blood glucose concentrations were determined every 5 min using a Contour Next glucometer (BAYER).

### Ethics Statement

Animal studies were conducted in accordance with the Guide for the Care and Use of Laboratory. Animals published by the National Institutes of Health (Publication No.85-23, revised 1996). The study protocols were approved by the University of Virginia Animal Care & Use Committee.

### Statistical Analysis

All data are presented as mean ± SEM. Statistical analyses were performed with SigmaStat 11.0 software (Systat Software, Inc), using either Student’s *t* test or ANOVA with post-hoc analysis as appropriate. A *p*-value of <0.05 was considered statistically significant.

## Results

### GLP-1 and Exendin-4 Enter Various Brain Regions Rapidly via a GLP-1 Receptor-Mediated Process

GLP-1 and its analogs are known to enter into the central nervous system from peripheral circulation. We first examined whether GLP-1 receptors are required for GLP-1 and/or GLP-1 analog entry into various brain regions (Figure 1A). As shown in Figures 1B,C, both labeled GLP-1 (GLP-1-FAM) and exendin-4 (exendin-4-FAM) were detected in various brain regions (cerebellum, cortex, hippocampus, hypothalamus, and brain stem) 10 min after being infused into the peripheral circulation. Infusion of exendin-(9-39), a GLP-1 receptor antagonist, markedly decreased the amount of GLP-1-FAM fluorescence and almost completely abolished exendin-4-FAM fluorescence in various brain regions. Consistent with these findings, we found that GLP-1 receptors are abundantly expressed in all brain regions studied (Figure 1D).

### GLP-1 Infusion Increases PKA Activity in Various Brain Regions in Both Normal and Vagotomized Rats

**FIGURE 2:**
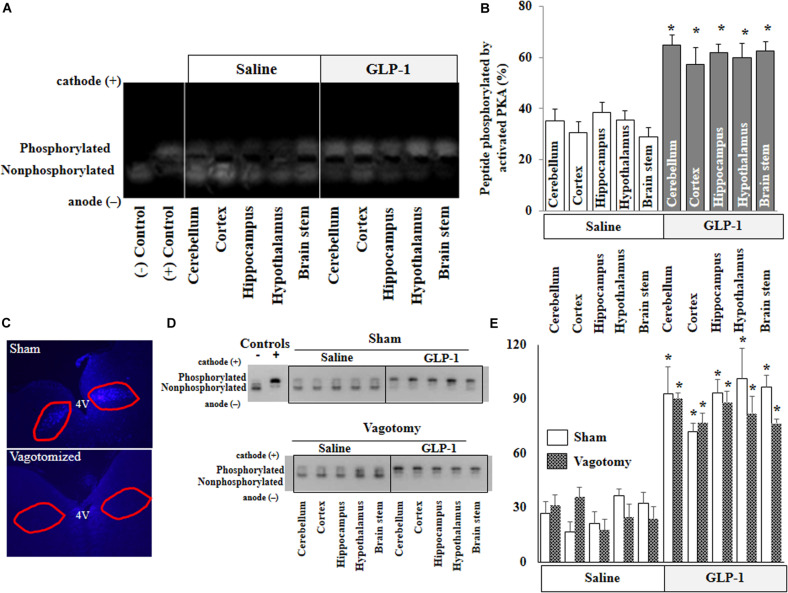
GLP-1 infusion increases PKA activity in various brain regions in both normal and vagotomized rats. Each rat received either saline or GLP-1 (30 pmol/kg/min) infusion via a carotid artery catheter for 10 min. PKA activities in various brain regions were determined in normal rats **(A,B)** and vagotomized rats **(D,E)**. **(C)** Fluorogold stain of dorsal motor vagal nucleus. Lack of staining in vagotomized rats confirms surgical completeness of vagotomy. **(A,D)**, PKA activity. **(B,E)**, Quantitative analysis (**B**, *n* = 7; *p < 0.05 vs. saline infusion, Student’s *t* test) (**E**, *n* = 6; *p < 0.05 vs. saline infusion, Student’s *t* test).

PKA is a key signaling intermediate in the GLP-1 receptor signaling pathway. To confirm that peripherally infused GLP-1 exerts biological effects in the brain, we determined PKA activities in various brain regions. PKA activities were significantly increased in all brain regions after peripheral infusion of GLP-1 (*p* < 0.05) but not after saline infusion for 10 min (Figures 2A,B). Sub-diaphragmatic resection of the vagus nerve, with surgical completeness confirmed by fluorogold label (Figure 2C) and lack of weight gain (Supplementary Figure 2), had no effects on GLP-1-induced acute activation of PKA in the brain (Figures 2D,E).

### GLP-1 Does Not Acutely Enter CSF but Increases PKA Activity in Cerebral Microvessels

**FIGURE 3:**
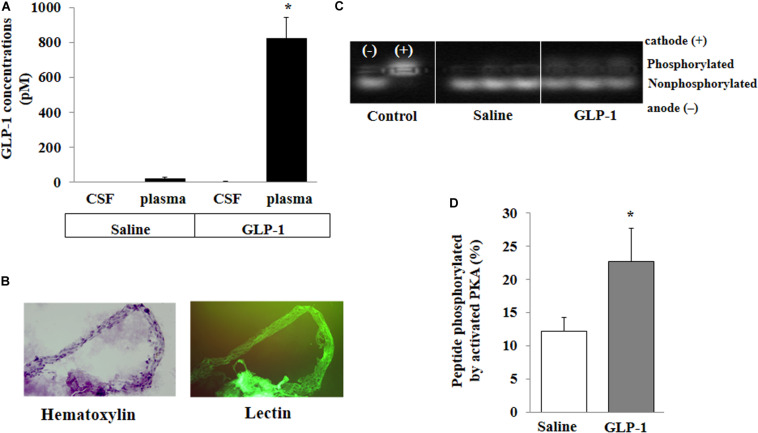
GLP-1 does not acutely enter cerebral spinal fluid (CSF) from circulation but activates PKA activity in cerebral microvessels. Each rat received a systemic infusion of either saline or GLP-1 (30 pmol/kg/min) for 10 min, GLP-1 concentrations in CSF and plasma were determined and cerebral microvessels were isolated. **(A)** GLP-1 concentrations in CSF and plasma (*n* = 4; *p < 0.01 vs. other groups, Student’s *t* test). **(B)** Isolated cerebral microvessels with Hematoxylin counterstain (left panel) or lectin stain (right panel). **(C)** PKA activity in isolated cerebral microvessel. **(D)** Quantitative analysis of PKA activity (*n* = 6, *p < 0.05, Student’s *t* test).

For peripherally infused GLP-1 to enter into the brain parenchyma, it could enter the CSF first and then go into the brain parenchyma or enter directly via microcirculation. We determined GLP-1 levels in the plasma and CSF. The levels of GLP-1 were very low in the plasma and undetectable in the CSF in saline-infused rats. Expectedly, systemic infusion of GLP-1 (30 pmol/kg/min) for 10 min markedly increased plasma GLP-1 concentrations. However, levels of GLP-1 remained undetectable in CSF (Figure 3A). On the other hand, GLP-1 acutely increased PKA activity in brain microvessels (*p* < 0.05) (Figures 3B–D).

### GLP-1 Receptors Are Abundantly Expressed in Various Blood Vessels, RBMECs, and Astrocytes

**FIGURE 4:**
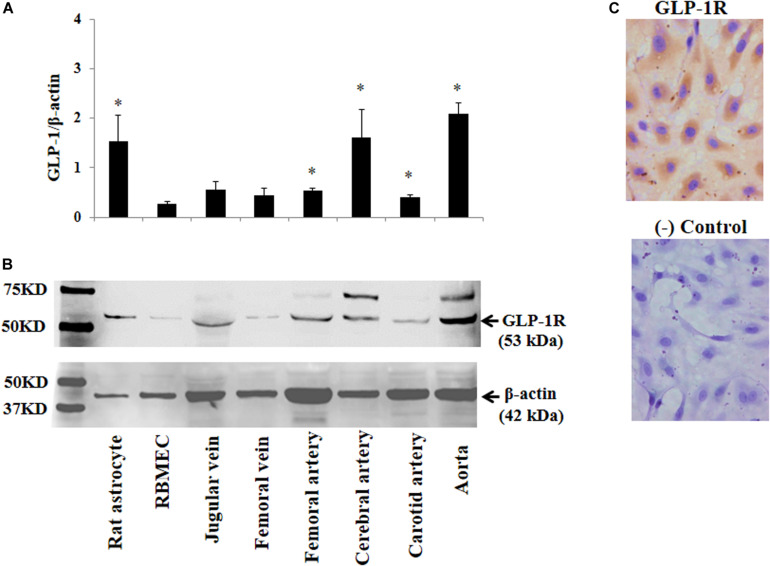
Expression of GLP-1 receptor in various blood vessels, RBMECs, and astrocytes. **(A)** Quantitative analysis of Western blot determination of GLP-1 receptor expression in cultured cells and isolated vessels (*n* = 5–7, *p < 0.05 vs. RBMECs, Student’s *t* test). **(B)** Representative Western blot images. **(C)** Immunocytochemical staining of GLP-1 receptor in cultured RBMECs. Negative control was done by omitting the primary antibody. GLP-1R – GLP-1 receptor.

The above findings led us to ask whether GLP-1 acutely enters brain via cerebral microvessels through a receptor dependent mechanism. We thus determined GLP-1 receptor expression in RBMECs, astrocytes, and cerebral arteries, along with other vessels such as jugular vein, femoral artery, carotid artery and aorta. As demonstrated by Western blot analysis in Figures 4A,B, GLP-1 receptors were expressed in all samples with astrocyte, cerebral artery and aorta having the highest levels of expression *(p* < 0.05). Cultured RBMECs also possess appreciable amount of GLP-1 receptors as evidenced by positive immunocytochemical staining (Figure 4C).

### RBMECs Actively Take Up and Transport GLP-1

**FIGURE 5:**
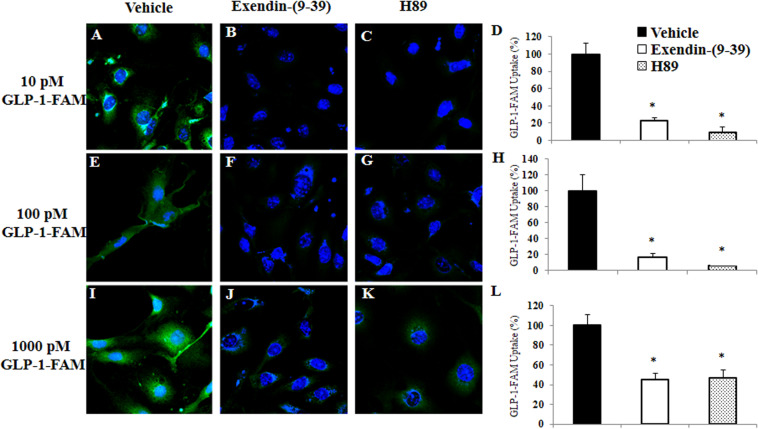
GLP-1 uptake by RBMECs is GLP-1 receptor and PKA dependent. RBMECs were pretreated with vehicle (PSS buffer, **A,E,I**), Exendin 9-39 (10 nM, **B,F,J**) or H89 (10 μM, **C,G,K**) for 30 min. Cells were then incubated with GLP-1-FAM at 10, 100 pM or 1 nM for 3 min. DAPI was used to stain the nucleus. Images were captured using confocal microscope. Fluorescence density was analyzed using ImageJ. **(D,H,L)** Quantitative analysis (*n* = 3, *p < 0.01, vs. vehicle, Student’s *t* test). GLP-1R – GLP-1 receptor.

**FIGURE 6:**
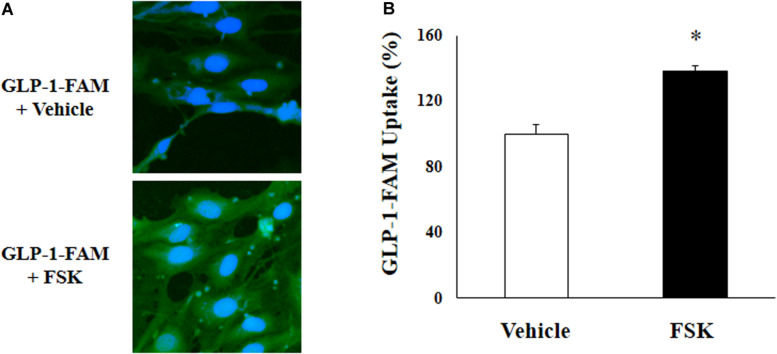
cAMP agonist increases GLP-1 uptake by RBMECs. **(A)** RBMECs were incubated with GLP-1-FAM at 10 pM for 3 min with or without 10 μM forskolin. DAPI was used to stain the nucleus. Images was captured using confocal microscope. Fluorescence density was analyzed using ImageJ. **(B)** quantitative analysis of the images (*n* = 6, *p < 0.05, vs. vehicle, Student’s *t* test).

To examine whether RBMECs actively take up GLP-1 and then transport it into the brain parenchyma, we first incubated RBMECs with different concentrations of labeled GLP-1 (GLP-1-FAM) (Figure 5). At all concentrations examined, RBMECs actively took up GLP-1-FAM (panels A, E, and I) and this action was blunted by either GLP-1 receptor antagonism with exendin-(9-39) (panels B, F, and J) or PKA inhibition with H89 (panels C, G, and K). Conversely, incubation of RBMECs with cAMP agonist forskolin significantly enhanced RBMEC uptake of GLP-1-FAM (Figure 6). Importantly, RBMECs did not take up free FAM molecule at 37°C or GLP-1-FMA at 4°C (Supplementary Figure 2). These data suggest that RBMECs actively take up GLP-1 via a GLP-1 receptor- and PKA-dependent mechanism.

**FIGURE 7:**
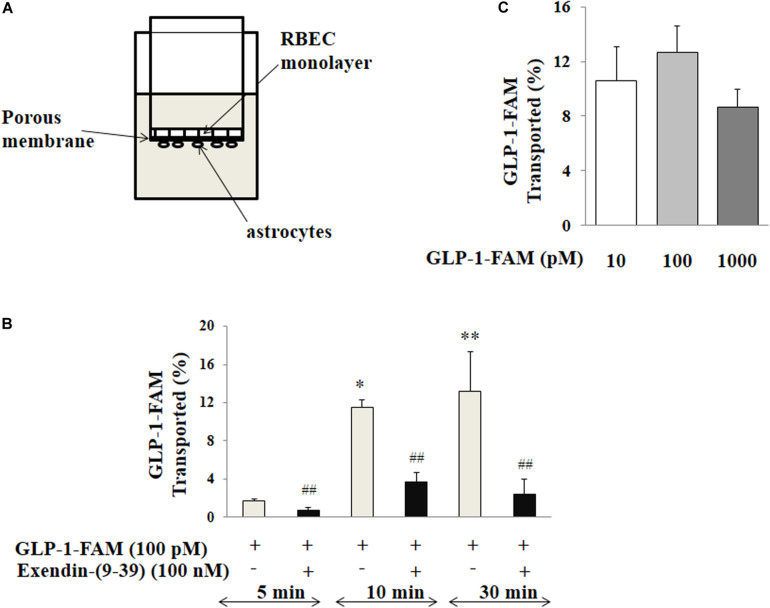
Trans-endothelial transport of GLP-1 is a GLP-1 receptor-mediated process. **(A)** Illustration of trans-well apparatus. **(B)** Time course of GLP-1-FAM transport across the RBMEC and astrocyte barrier, without (*n* = 7, *p < 0.05, **p < 0.01 vs. 5 min. One way ANOVA with Tukey’s post hoc analysis) or with exendin-(9-39) pretreatment (*n* = 7, ^##^*p* < 0.01 vs. GLP-1-FAM at each time point, Student’s *t* test). **(C)** Dose-response of GLP-1-FAM transport across the RBMEC and astrocyte barrier after 10 min (*n* = 6, *p* = 0.37, One way ANOVA). Inulin-FITC was used to determine the passive “leak” through the artificial BBB. GLP-1-FAM or inulin-FITC transport to the lower chamber was determined by measuring FAM and FITC fluorescence in the lower chamber. GLP-1R – GLP-1 receptor.

We next assembled an artificial BBB consisting of an endothelial and an astrocyte layer and examined whether GLP-1 crossed from one side to the other (Figure 7A). GLP-1-FAM crossed the barrier in a time-dependent fashion and was clearly blunted by GLP-1 receptor antagonism (Figure 7B). The lack of significant difference between physiological (10 and 100 pM) and pharmacology (1000 pM) concentrations suggests that the tran-endothelial transport of GLP-1-FAM does not appear to be concentration dependent (Figure 7C), suggesting endothelial transport of GLP-1 is a regulated process.

### GLP-1 Does Not Acutely Affect Hippocampal Microvascular Perfusion

**FIGURE 8:**
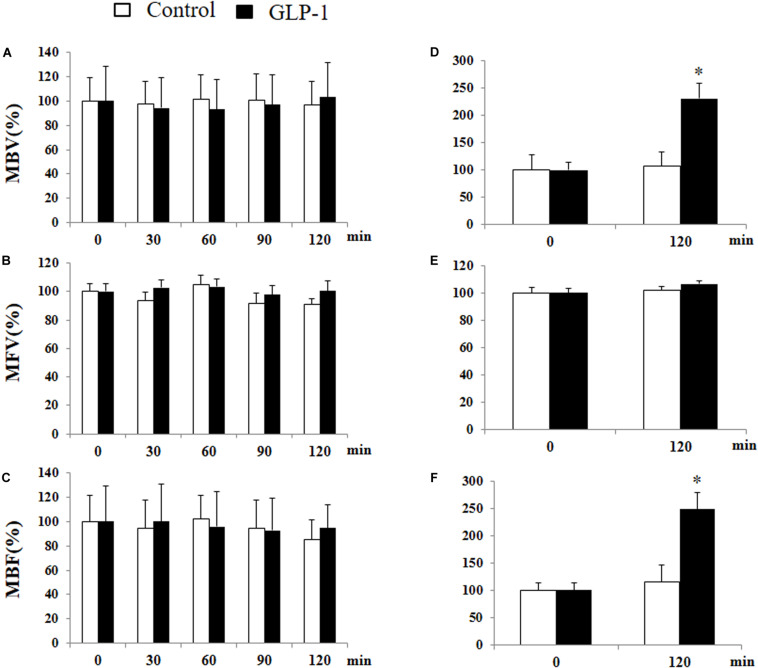
GLP-1 infusion increases muscle but not hypothalamic microvascular perfusion. Each rat received a systemic infusion of saline or GLP-1 at 30 pmol/kg/min for 120 min and MBV, MFV and MBF were determined using contrast-enhanced ultrasound in both hypothalamus **(A,B,C)** and muscle **(D,E,F)** (*n* = 6, *p < 0.05, vs. control, Student’s *t* test).

We have previously shown that GLP-1 has a profound vasodilatory action in skeletal muscle microvasculature and this action is associated with increased muscle delivery of insulin and oxygen, via a PKA-mediated pathway (Chai et al., 2012, 2014; Dong et al., 2013). Given the significant activation of PKA in cerebral microvessels after 10 min of GLP-1 infusion, we finally examined the effect of GLP-1 on hippocampal microvascular perfusion. We also measured GLP-1-mediated microvascular perfusion in muscle as a positive control. GLP-1 infusion for 120 min did not alter hippocampal MBV, MFV, or MBF (Figure 8). However, it did, similar to our prior findings (Chai et al., 2012, 2014; Dong et al., 2013), markedly increase both MBV and MBF in muscle (p < 0.05) without affecting MFV. Blood glucose and MAP remained stable during the study (Supplementary Figure 4).

## Discussion

Diabetes is associated with an increased risk of cognitive impairment, dementia, and stroke in addition to macro- and microvascular complications (Bornstein et al., 2014). While the underlying mechanisms remain to be defined, changes to brain vasculature, insulin resistance, oxidative distress and alterations in amyloid metabolism have all been implicated (Bornstein et al., 2014). GLP-1 and its analogs have been shown to exert multiple salutary central actions that benefit patients with diabetes. The current results demonstrated that peripherally derived GLP-1 and its analog exendin-4 can rapidly enter various regions of brain via a GLP-1 receptor-dependent pathway and this process is actively regulated via endothelial GLP-1 receptors in the brain microvasculature.

The central actions of GLP-1 and its analogs have been well recognized. GLP-1 analogs have been repeatedly shown to cross the BBB, stimulate brain GLP-1 receptors, and modulate mitochondrial function, protein aggregation, neuroinflammation, synaptic plasticity, learning and memory in experimental models of Parkinson’s and Alzheimer’s disease (Athauda and Foltynie, 2016). Our observation that peripherally administrated GLP-1 and exendin-4 rapidly enter various brain regions is consistent with prior findings that peripherally injected GLP-1 analogs can easily enter the brain (Kastin and Akerstrom, 2003; Secher et al., 2014). This suggests that acute elevation of plasma GLP-1 levels as seen postprandially or after administration of the GLP-1 analogs can rapidly act on various regions of the brain to exert biological actions. Indeed, intravenous injected exendin-4 can be detected in the brain within 5 min and the appearance is time-dependent in the first 25 min (Kastin and Akerstrom, 2003) and we show here that this rapid entry into the brain is associated with biological activity in these brain regions as evidenced by increased PKA activities. The lack of GLP-1 in detectable amount in CSF at a time of rapid activation of PKA in brain parenchyma suggests that GLP-1 did not enter the brain via the blood-CSF barrier which has fenestrated capillaries at the choroid plexus (Gray and Barrett, 2018). It is possible that the accumulation of GLP-1 or exendin-4 inside the microvessel endothelium may have contributed to the observed increase in fluorescence and PKA activity in the brain regions. However, these changes most likely reflected such accumulation in the brain tissues given that neurons and glial cells markedly outnumber brain endothelial cells and that the *in vitro* study showed that GLP-1 rapidly passes through the endothelium.

GLP-1 receptors are expressed in multiple brain areas in rodents, primates, and humans and they are involved in learning, neuroprotection and regulation of appetite and satiety (Wei and Mojsov, 1995; During et al., 2003; Richards et al., 2014). Global knockout of the GLP-1 receptor in mice impairs synaptic plasticity and memory formation (Abbas et al., 2009). While peptides can cross BBB via either constitutive vesicular movement or regulated transport, our results suggest that GLP-1 receptor plays a regulatory role in the case of GLP-1 or its analog. Indeed, pre-treatment of animals with GLP-1 receptor antagonist exendin (9-39) blunted GLP-1 and exendin-4 entry into the brain. Furthermore, incubation of endothelial cells with exendin-(9-39) inhibited endothelial uptake and trans-endothelial transport of labeled GLP-1. That inhibition of PKA with H89 blocks while stimulation of PKA with forskolin, which activates adenylate cyclase and increases cAMP levels, enhances endothelial uptake of GLP-1 argues strongly that GLP-1 receptor activity plays important role in facilitating GLP-1 entry into the brain. The lack of concentration dependence further supports that this is a regulated process. We noted that pre-treatment of the animals with exendin-(9-39) nearly completely blocked exendin-4-FAM entry into the brain but only partially so for labeled GLP-1. Likely rapid degradation of GLP-1-FAM in plasma by DPP4 (half-life of GLP-1 ∼2 min) is contributing GLP-1-FAM degradation products (including free FAM) to plasma fluorescence signal and these products are not taken up by the GLP-1 receptor. By comparison, exendin-4 is DPP4 resistant and has a half-life of 1.5 h.

Previous study has shown that vagal afferent neuron GLP-1 receptors play a crucial role in mediating the effects of GLP-1 on food intake and glycemia (Krieger et al., 2016). While a full expression of vagal afferent neuron GLP-1 receptors is not necessary for the maintenance of long-term energy balance in normal eating conditions, vagal afferent neuron GLP-1 receptor knockdown increases meal size, accelerates gastric emptying, and blunts post-meal insulin release (Krieger et al., 2016). To assess the role of the vagus nerve on GLP-1 and its analog entry into the brain, we infused GLP-1 peripherally to rats with sub-diaphragmatic resection of the vagal nerve. The completeness of the vagal resection was confirmed by fluorogold staining and a lack of weight gain (Supplementary Figure 2). Similar to normal rats, we observed a rapid and robust increase in PKA activity in various brain regions in both sham-operated and vagotomized rats. This confirms that GLP-1 entry into the brain is independent of vagal nerve.

GLP-1 rapidly activates endothelial nitric oxide synthase and causes vasodilation. We have previously shown that it potently dilates pre-capillary arterioles to increase microvascular perfusion in the skeletal muscle via a nitric oxide- and PKA-dependent mechanism (Chai et al., 2012, 2014; Dong et al., 2013; Subaran et al., 2014; Tan et al., 2018). In the current study, we also observed that GLP-1 robustly increased muscle microvascular perfusion but interestingly GLP-1 infusion did not alter microvascular perfusion in the hypothalamus. This is in contrast to insulin which also is a vasodilatory hormone and we and others have shown it increases microvascular perfusion in both the skeletal muscle (Barrett et al., 2009, 2011; Barrett and Liu, 2013) and hypothalamus (Fu et al., 2017), and of surprise as GLP-1 infusion enhanced PKA activity in cerebral microvessels and we demonstrated that in skeletal muscle GLP-1 enhances microvascular perfusion via PKA-mediated pathway (Dong et al., 2013). Whether GLP-1 affects microvascular perfusion in other parts of the brain remains to be studied. Given that hypothalamus expresses abundant GLP-1 receptors, administration of GLP-1 acutely increased PKA activity and there is no increase in hypothalamic microvascular perfusion, it appears that peripherally derived GLP-1 can effectively reach hypothalamic parenchyma without expanding the microvascular blood volume, which is important in insulin delivery in skeletal muscle (Barrett et al., 2009, 2011).

In conclusion, our data demonstrate that peripherally administrated GLP-1 and its analog exendin-4 can rapidly enter the brain and brain microvascular endothelial cells are able to actively take up and transport GLP-1. These processes are dependent on an active ligand binding to and activating the GLP-1 receptor. These findings suggest that peripherally derived GLP-1 or administered GLP-1 agonists can acutely act on the brain to exert biological effects and argue also for the possibility of using GLP-1 or its analog as shuttle peptide to deliver medications directly to the brain parenchyma.

## Data Availability Statement

The datasets generated for this study are available on request to the corresponding author.

## Ethics Statement

The animal study was reviewed and approved by University of Virginia Animal Care and Use Committee.

## Author Contributions

ZF, LG, JL, KA, and ZL researched data. JW and EB contributed to study design and discussion. ZF, LG, JL, and ZL wrote the manuscript.

## Conflict of Interest

The authors declare that the research was conducted in the absence of any commercial or financial relationships that could be construed as a potential conflict of interest.

## Footnotes

1 https://imagej.nih.gov/ij/
