# Supplementary material for: Brain Endothelial Cells Regulate Glucagon-Like Peptide 1 Entry Into the Brain via a Receptor-Mediated Process
Source: Front Physiol. 2020 May 29;11:555. doi: 10.3389/fphys.2020.00555 (PMC7274078; doi:10.3389/fphys.2020.00555)
Supplement: Supplementary file 1 [file Data_Sheet_1.PDF]

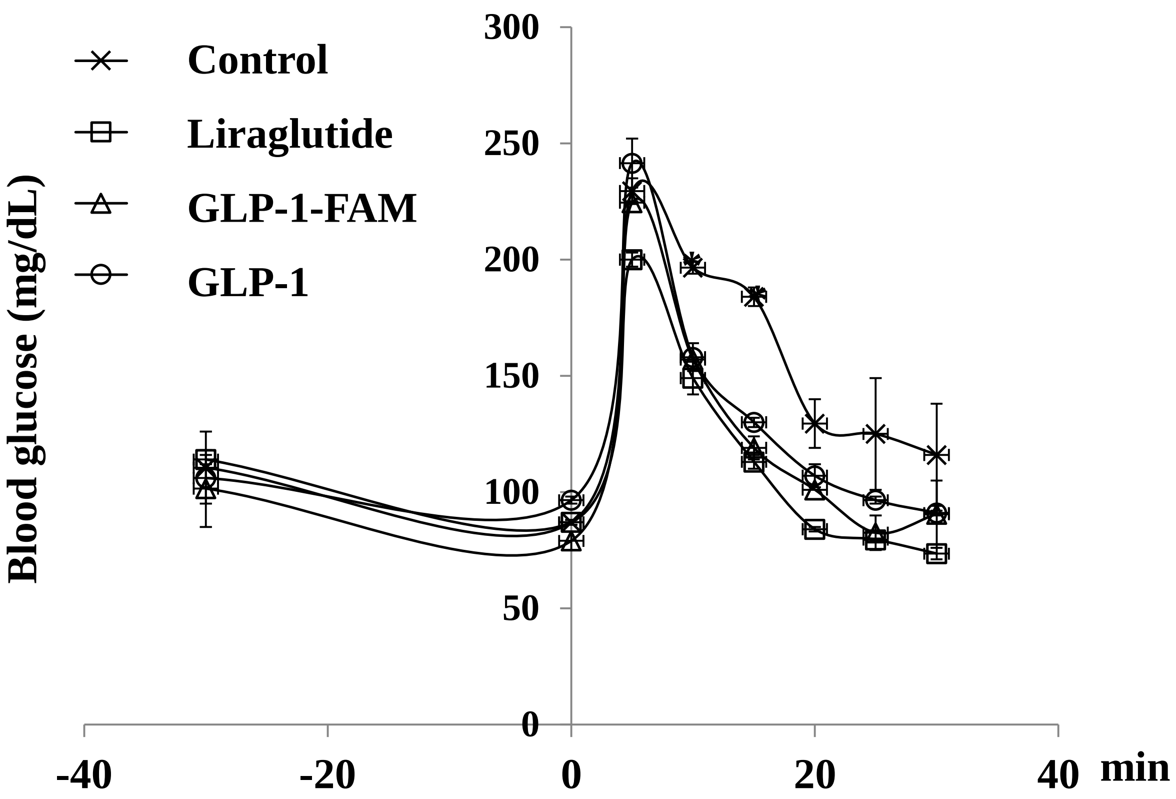

Supplemental Fig. 1. Intravenous infusion of GLP-1-FAM lowers rat blood glucose during glucose tolerance test (0.5/kg glucose)**.** Biological function of GLP-1-FAM (30 pmol/kg/min) was compared with liraglutide (30 pmol/kg, i.v.) and GLP-1(30 pmol/kg/min) (n=2, * $p<0.05$).

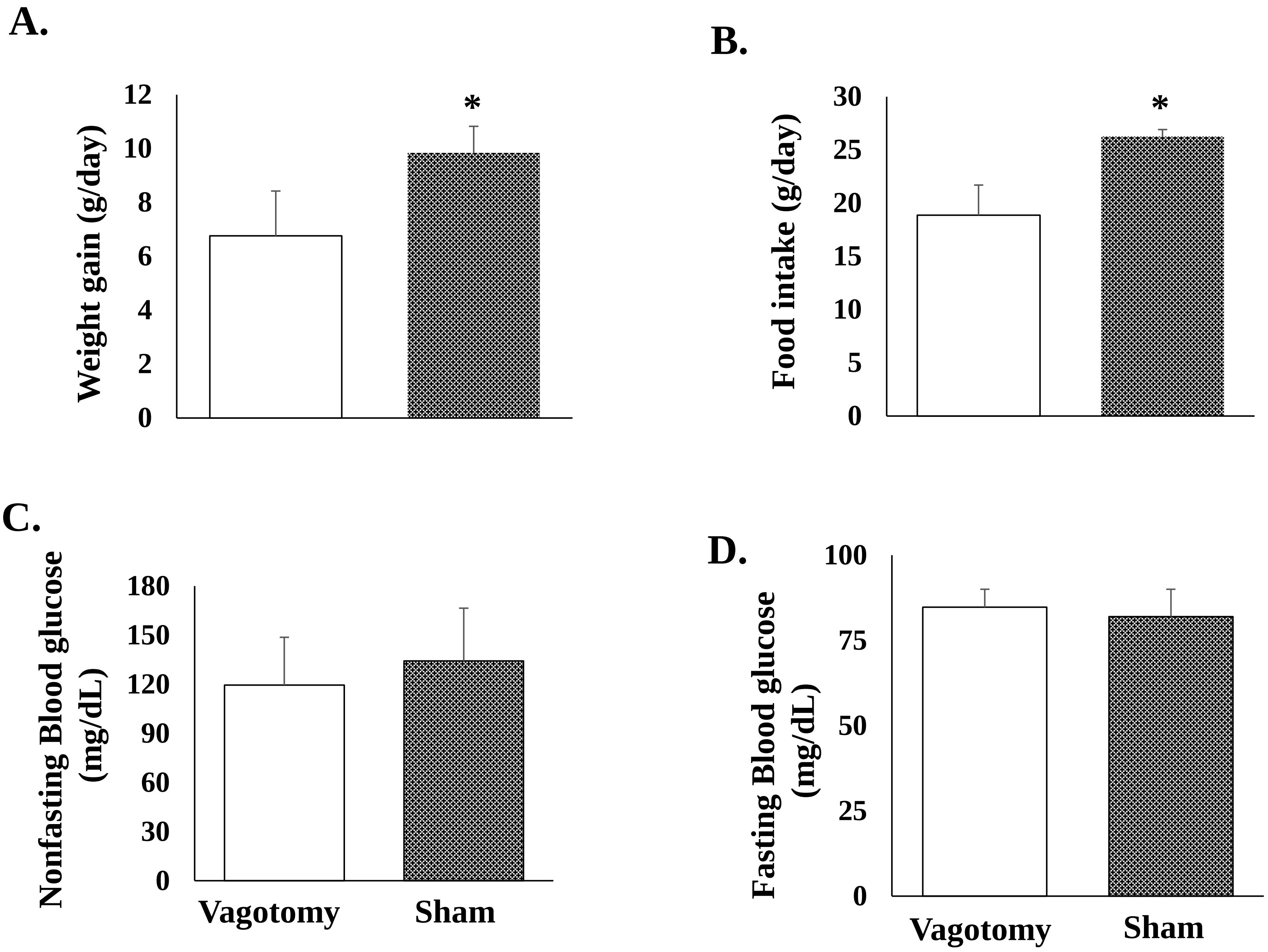

Supplemental Fig. 2. Daily body weight gain (A), daily food intake (B), nonfasting blood glucose (C) and fasting blood glucose (D) in vagotomized and sham operated rats. (n=5 each)

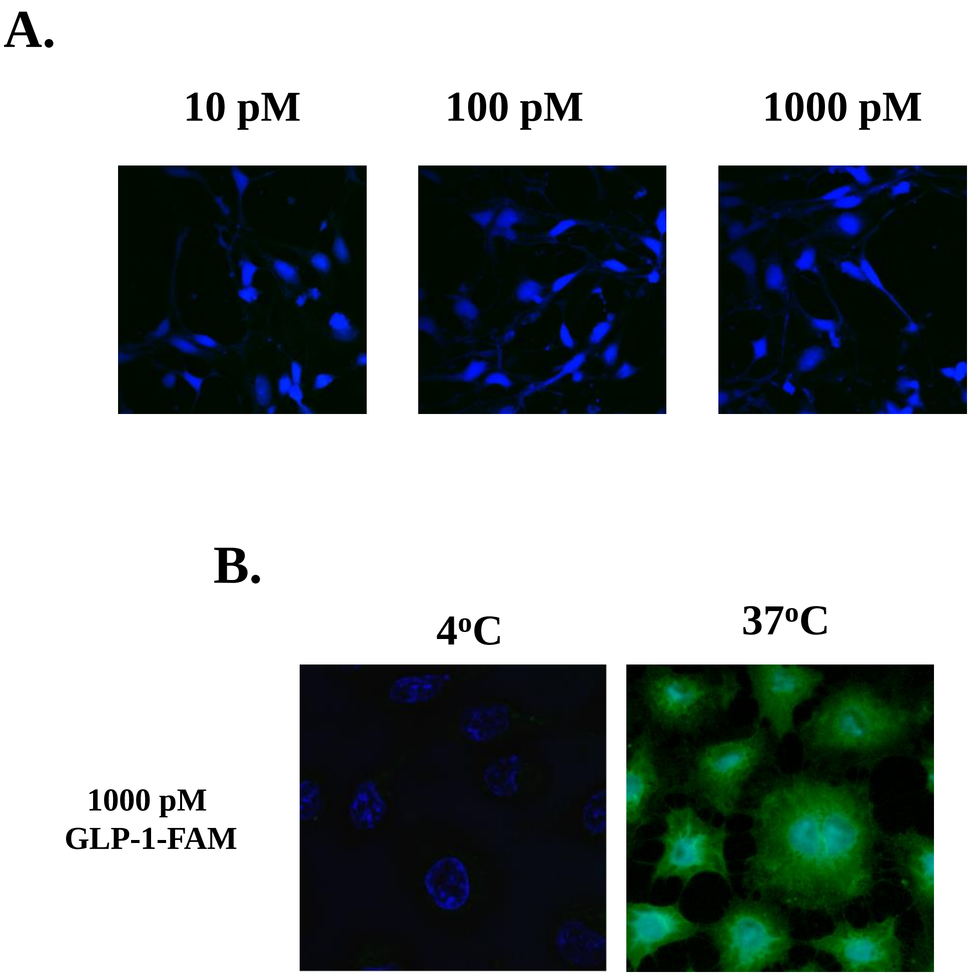

Supplemental Fig. 3. RBMECs do not uptake free FAM molecule at 37°C (A) or GLP-1-FAM at 4°C (B). RBMECs were incubated with FAM (10, 100, or 1000 pM) at 37°C or GLP-1-FAM (1000 pM) at 4 °C or 37°C for 3 min. DAPI was used to stain the nucleus. Images was captured using confocal microscope.

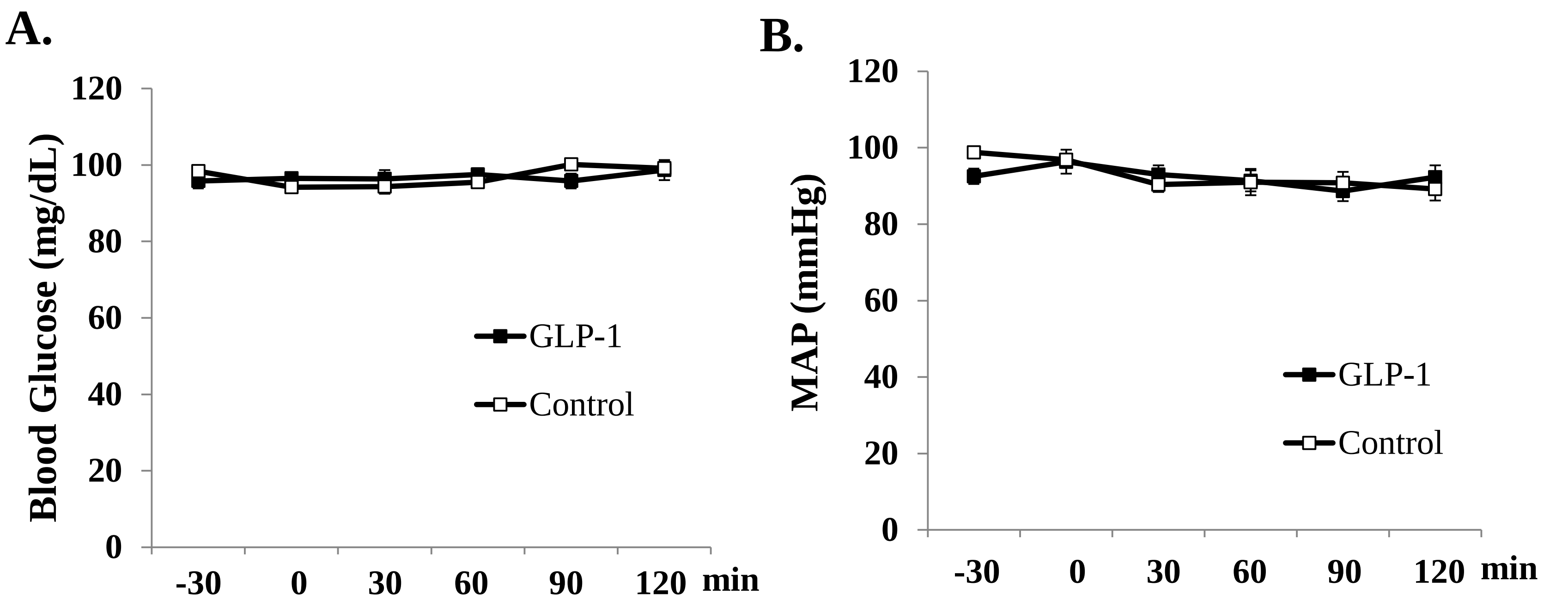

Supplemental Fig. 4. Blood glucose (A) and blood pressure (B) during GLP-1 or saline (control) infusion (n=6).
